# Comparative evaluation of point-of-care blood gas analysis in rabbit disease models

**DOI:** 10.3389/fvets.2025.1621912

**Published:** 2025-06-27

**Authors:** Soo Jeong Park, Seong Eun Cho, Tran Xuan Ngoc Huy, Suk Kim, Sang Hun Kim, Beomjin Park

**Affiliations:** ^1^Institute of Animal Medicine, College of Veterinary Medicine, Gyeongsang National University, Jinju, Gyeongsangnam-do, Republic of Korea; ^2^Hanaro Veterinary Clinic, Daedong, Daejeon, Republic of Korea; ^3^Department of Information and Statistics, Gyeongsang National University, Jinju, Gyeongsangnam-do, Republic of Korea

**Keywords:** blood gas analysis, reference interval, rabbit, disease models, acid-base, electrolyte

## Abstract

**Objective:**

This study aims to evaluate the blood gas results in various rabbit disease models and to establish the reference interval of venous and arterial blood gas parameters for clinically healthy and diseased rabbits. The primary purpose of this study is to evaluate various disease effects on acid-base and electrolyte disturbance and to examine the clinical value of blood gas analysis in rabbits.

**Methods:**

Two hundred rabbits with various breeds were included in the study. Rabbits were divided into dental, gastrointestinal, musculoskeletal, and urologic disease model groups and one clinically healthy group. Venous and arterial blood gas analyses were performed with a point-of-care blood gas analyzer, i-Smart 300 VET Blood Gas Analyzer (i-SENSE, Korea). The reference intervals for the blood gas results were established. Reference intervals for each diseased group were compared with a clinically healthy control group to analyze the disease's effect on blood gas values.

**Results:**

Several acid-base and electrolyte derangements were confirmed. The dental disease model had lower pH (power of hydrogen), PCO_2_ (partial pressure of carbon dioxide), and HCO_3_ (bicarbonate) than the control group (*p*-values < 0.001 by *t*-tests for both pH and HCO_3_, and *p*-value < 0.01 by a *t*-test for arterial PCO_2_). The gastrointestinal disease model demonstrated hyponatremia and hypocalcemia compared to the control group, with the *p*-values < 0.01 by *t*-tests for both sodium and calcium. The musculoskeletal disease model had lower Hct (hematocrit) and HGB (hemoglobin) than the control (*p*-values < 0.01 by *t*-tests for both Hct and HGB). The urologic disease group did not have a statistically significant difference in the reference interval of blood gas results.

**Conclusions:**

The results indicated that various disease models in rabbits alter blood gas values. Diseases in body systems correlated with acid-base and electrolyte regulation are more likely to induce blood gas derangements. However, blood gas evaluation in various rabbit diseases is limited. Further studies with more cases in equivalent distribution and other disease models are needed.

**Clinical relevance:**

This study is the first to present the reference interval for venous and arterial blood gas results in various disease models of rabbits. Also, this study presented the clinical value of point-of-care blood gas analysis in rabbit diseases. Blood gas analysis has potential diagnostic and prognostic value on various rabbit diseases.

## 1 Introduction

Blood gas analysis is routinely employed in veterinary medicine to evaluate physiological parameters such as acid-base status and electrolyte balance, whose imbalances can indicate underlying disease processes that require immediate clinical attention (1, 2). Critical electrolytes, including sodium, chloride, potassium, and calcium, assessed through blood gas analysis, are essential for maintaining physiological homeostasis, including fluid balance and cellular electrochemical gradients; imbalances in these parameters can pose severe risks and necessitate prompt clinical intervention (2).

Blood gas analyses can be performed using either venous or arterial blood samples, each reflecting distinct physiological states and diagnostic utility. Venous blood primarily reflects tissue-level oxygenation and cellular metabolic status, making it particularly suitable for assessing acid-base balance at the cellular level and evaluating tissue perfusion. In contrast, arterial blood analysis remains the golden standard for accurately assessing oxygenation and pulmonary ventilation, providing precise measurements of partial pressure of oxygen (PO_2_), partial pressure of carbon dioxide (PCO_2_), and oxygen saturation of hemoglobin (SO_2_) (3, 4). Consequently, arterial blood samples are routinely employed in clinical settings to monitor patients with respiratory disorders or those undergoing anesthesia (2).

The pH measurement in blood gas analysis indicates the severity of acid-base disturbances, with a normal reference value around 7.4 in humans, dogs, and rabbits (4). Classification of acid-base disorders as metabolic, respiratory, or mixed relies on evaluating bicarbonate (HCO_3_) and PCO_2_, facilitating identification of primary etiologies and compensatory mechanisms. Respiratory disturbances typically arise from dysfunction in the respiratory system or related neurological, muscular, or hematologic disorders, as the respiratory system primarily regulates homeostasis by controlling the levels of PO_2_ and PCO_2_ through pulmonary gas exchange. Conversely, metabolic disturbances generally stem from gastrointestinal, urinary, hepatic, or endocrine pathologies because electrolyte and pH homeostasis is predominantly maintained by electrolyte absorption and excretion via the gastrointestinal and urinary systems (3).

Rabbits often exhibit non-specific clinical signs and are particularly vulnerable to electrolyte disturbances due to limited compensatory renal function and complex gastrointestinal physiology (5). Additionally, blood parameters in rabbits are influenced by breed, sex, and age (5). Despite the widespread utilization of rabbits in laboratory research, most reported blood gas reference values derive from specific biomedical models without adequate consideration of breed, sex, or underlying disease (4). Studies conducted on clinically healthy New Zealand White rabbits constitute a significant portion of existing literature (6). Venous blood gas analysis has been performed in rabbits experiencing specific conditions such as pregnancy (7), pneumoperitoneum (8), and gastric stasis or dilation (9). Arterial blood gas studies in conscious rabbits (10) and those under anesthesia (11) or mechanical ventilation (12) have underscored the value of this method in evaluating respiratory function.

Comprehensive blood gas data across various rabbit breeds, compared to other companion species such as dogs and cats, remain scarce (4–6). Research explicitly evaluating the influence of diseases on both arterial and venous blood gas parameters is notably limited. Furthermore, established reference intervals for blood gas variables vary among veterinary species (6), highlighting the necessity of tailored reference standards for rabbits.

The present study aims to enhance the clinical applicability of blood gas analysis in rabbits by examining both healthy and clinically diseased subjects, thereby assessing the impact of gastrointestinal, urologic, dental, and musculoskeletal diseases on acid-base and electrolyte parameters. Additionally, this research aims to establish robust reference intervals for blood gas measurements, taking into consideration potential variability in these parameters associated with clinical factors, including sex and blood sample type.

Conducted on a substantial sample size comprising 200 rabbits encompassing diverse breeds, sexes, blood sample types, and clinical disease categories, this study seeks to deliver comprehensive clinical assessments. These comparative evaluations and the generated reference intervals reflecting multiple clinical factors will provide foundational data crucial for the development of laboratory-based diagnostic criteria and prognostic markers in rabbit medicine.

## 2 Materials and methods

### 2.1 Study design and animals

A total of 200 domestic rabbits (126 males, 74 females), weighing between 1.5 and 4.5 kg, were admitted to Hanaro Veterinary Clinic (Daejeon, Republic of Korea) from August 1, 2024, to January 31, 2025. Breeds were selected based on their prevalence in research and common usage as pets. The breeds included Mongrel, Lionhead, Lop Eared, Mixed, Dwarf, Dutch, Rex, and New Zealand White rabbits. Mongrels were most frequently represented (*n* = 72), followed by Lionheads (*n* = 65), Lop Eared (*n* = 22), Mixed breeds (*n* = 18), and Dwarfs (*n* = 10).

Rabbits were classified into five groups: one clinically healthy control group (Normal) and four groups with clinically evident diseases. The disease groups included gastrointestinal (GI), dental (Dn), musculoskeletal (Mus), and urologic (Uro) conditions. The detailed distribution of rabbits according to disease group, sex, and blood sample type is summarized in Table 1.

**Table 1:** Number of rabbits according to the disease group, sex, and blood sample.

**Sex**	**Blood sample**	
	**Venous**	**Arterial**
**Normal**		
Male	26	52
Female	15	36
**Dn**		
Male	7	14
Female	4	5
**GI**		
Male	6	12
Female	3	5
**Mus**		
Male	3	3
Female	2	1
**Uro**		
Male	0	3
Female	1	2
**Total**		
Male	42	84
Female	25	49

### 2.2 Sample collection

Rabbits were sedated using isoflurane administered via face mask to minimize stress and pain during the sampling procedure. Isoflurane sedation is routinely employed due to its minimal influence on blood gas and biochemical parameters, thus accurately reflecting the animal's physiological status at the time of sample collection (3, 4). Subsequently, anesthesia was induced in all rabbits using intravenous sodium pentobarbital (25 mg/kg) administered through the ear vein. The adequacy of anesthesia was confirmed by assessing the absence of a response to painful stimuli. Blood samples were then anaerobically collected from either the central auricular artery or marginal ear vein. A total volume of 0.5 ml of arterial or venous blood was obtained using a preheparinized syringe fitted with a 27-gauge needle and immediately processed for blood gas analysis. Following blood collection, rabbits were closely monitored to ensure complete recovery from sedation. No adverse effects were observed in any rabbits post-procedure.

### 2.3 Blood gas analysis

Blood gas analyses were performed using the i-Smart 300 VET Blood Gas Analyzer (i-SENSE, Korea), a device recognized for its accuracy and reliability within veterinary practice (13). This point-of-care analyzer was chosen due to its widespread application in veterinary medicine and its capability to deliver prompt results (13). Calibration and quality control procedures were strictly followed according to the manufacturer's guidelines to ensure the accuracy and validation of the obtained measurements. Parameters evaluated are summarized in Table 2. Blood gas analyses were conducted immediately following blood collection, and results were available within minutes, allowing for rapid assessment of each rabbit's physiological condition.

**Table 2:** Evaluated blood gas parameters with corresponding symbols and measurement units.

**Parameter**	**Symbol**	**Unit**	**Parameter**	**Symbol**	**Unit**
Power of hydrogen	pH		Standard bicarbonate	HCO_3_-Std	mmHg
Partial pressure of carbon dioxide	PCO_2_	mmHg	Buffer base	BB	mmol/L
Partial pressure of oxygen	PO_2_	mmHg	Base excess	BE	mmol/L
Sodium	Na	mmol/L	Total carbon dioxide	TCO_2_	mmol/L
Potassium	K	mmol/L	Alveolar-arterial oxygen gradient	A-a gradient	mmHg
Calcium	Ca	mg/dL	Hemoglobin	HGB	g/dL
Chloride	Cl	mmol/dL	Oxygen saturation	SO_2_	%
Hematocrit	Hct	%	Anion gap	AG	mmol/L
Bicarbonate	HCO_3_	mmol/L	Ionized calcium	iCal	mmol/L

### 2.4 Statistical analysis

All statistical analyses were performed using R software version 4.4.1 (R Project for Statistical Computing), employing the *MASS* package (version 7.3-65) to perform Box-Cox transformations and the *referenceIntervals* (version 1.3.1) for the determination of reference intervals. Prior to statistical analysis, all collected data were thoroughly inspected to identify potential outliers, which were generally defined as data points significantly deviating beyond specific standard deviations from the mean. Any identified outliers were excluded from subsequent analyses. While the removal of outliers generally changes statistical results, their inclusion can introduce inaccuracies in the determination of reference intervals (14). To enhance the reliability and validity of the findings, Horn's method (14) was employed to detect multiple outliers within the dataset. Additionally, rabbit cases containing incomplete or missing values were also excluded from the analysis to prevent potential bias. Only cases with complete datasets were included in the final analysis.

#### 2.4.1 Identifying distributional differences

All measurements were collected with consideration of specific factors, including sex, blood sample type, and disease category. However, in the absence of significant distributional differences related to a given factor, combining data across related categories can enhance statistical power and precision in establishing reference intervals. To identify potential distributional differences in measurements, the two-sample Kolmogorov-Smirnov test was employed, comparing distributions across sexes and blood sample types within each disease group. The Kolmogorov-Smirnov test assumes that the measurements are independent and identically distributed samples drawn from continuous distributions (15). These assumptions, generally considered weak conditions, were met by all measurements in this study.

Due to the diverse measurements analyzed, multiple statistical tests were anticipated, increasing the possibility of type I errors. To mitigate this risk, the significance level was adjusted using the Bonferroni correction (16). Additionally, samples from rabbits with musculoskeletal and urologic diseases were excluded from this analysis because of insufficient sample sizes.

#### 2.4.2 Analysis of reference interval

Reference intervals for physiological measurements were established using samples obtained from normal, healthy rabbits. Based on preliminary analyses, specific parameters, including PCO_2_, PO_2_, TCO_2_, SO_2_, and HCO_3_, were analyzed separately for arterial and venous blood samples. A-a gradient was analyzed exclusively for arterial samples.

Reference intervals are commonly established through two statistical approaches: parametric and non-parametric. The parametric approach assumes that the data follow a specific probability distribution, commonly a normal distribution. This assumption is utilized to define the interval that contains a given probability, such as 0.95, under the assumed probability distribution. In contrast, the non-parametric approach derives the reference interval from sample quantiles, which are calculated based on the empirical distribution.

The non-parametric approach is advantageous because it does not require assumptions about the underlying distribution. However, it is generally recommended for use when the number of reference individuals within a group is large, typically at least 120 (17, 18). Moreover, if there is sufficient statistical evidence suggesting that the data adheres to a specific distribution, the parametric approach is more suitable because it provides asymptotically more efficient estimates compared to the non-parametric approach. Therefore, following the guidelines established in (18), the choice between parametric and non-parametric methods was based on the following criteria: the non-parametric approach was employed when the sample size is at least equal to 120; otherwise, the parametric approach is utilized if the data was found to be normally distributed by the Shapiro-Wilk test. If the data was not normally distributed, the data was transformed with a Box-Cox transformation. In instances where the Box-Cox transformation was ineffective, the robust method in (19) was applied.

#### 2.4.3 Comparison of reference intervals between healthy and disease groups

The reference intervals derived in this study were compared against intervals covering the majority of measurements obtained from diseased subjects. For the purpose of clarity and consistency with terminology previously established in (20), intervals determined from healthy populations are referred to as “healthy-associated reference intervals,” whereas those pertaining specifically to diseased groups are designated as “disease-associated reference intervals.” It is important to recognize, as previously highlighted in (21), that reference intervals may not inherently be optimal tools for disease diagnosis. By statistical design, ~5% (or 10%, depending upon the selected probability threshold) of clinically healthy individuals may fall outside established reference intervals. Moreover, established reference intervals do not account for the sensitivity necessary for the identification of specific diseases, potentially limiting their clinical utility in diagnostic applications. Nevertheless, comparative analysis between healthy-associated and disease-associated intervals offers valuable insights into disease-induced variations in specific parameters. Such analysis aids not only in enhancing differential diagnostic accuracy but also informs subsequent clinical management decisions and therapeutic interventions.

Disease-associated reference intervals were generated utilizing methodologies consistent with those employed for healthy populations. Furthermore, statistical comparisons were made between healthy and diseased groups by evaluating the 90% confidence intervals (CIs) of reference limits and conducting *t*-tests accompanied by Levene's test for homogeneity of variance to identify statistically significant differences.

## 3 Results

### 3.1 Identifying distributional differences

All measurements derived from arterial and venous blood samples across disease categories showed no statistically significant differences between sexes, as evaluated at a significance level of 0.05. Nonetheless, specific measurements exhibited statistically significant differences between arterial and venous samples when stratified by sex and disease category. Particularly, in data analysis from clinically healthy subjects, statistically significant differences were identified in the distributions of PCO_2_, PO_2_, and SO_2_.

**Figure 1:**
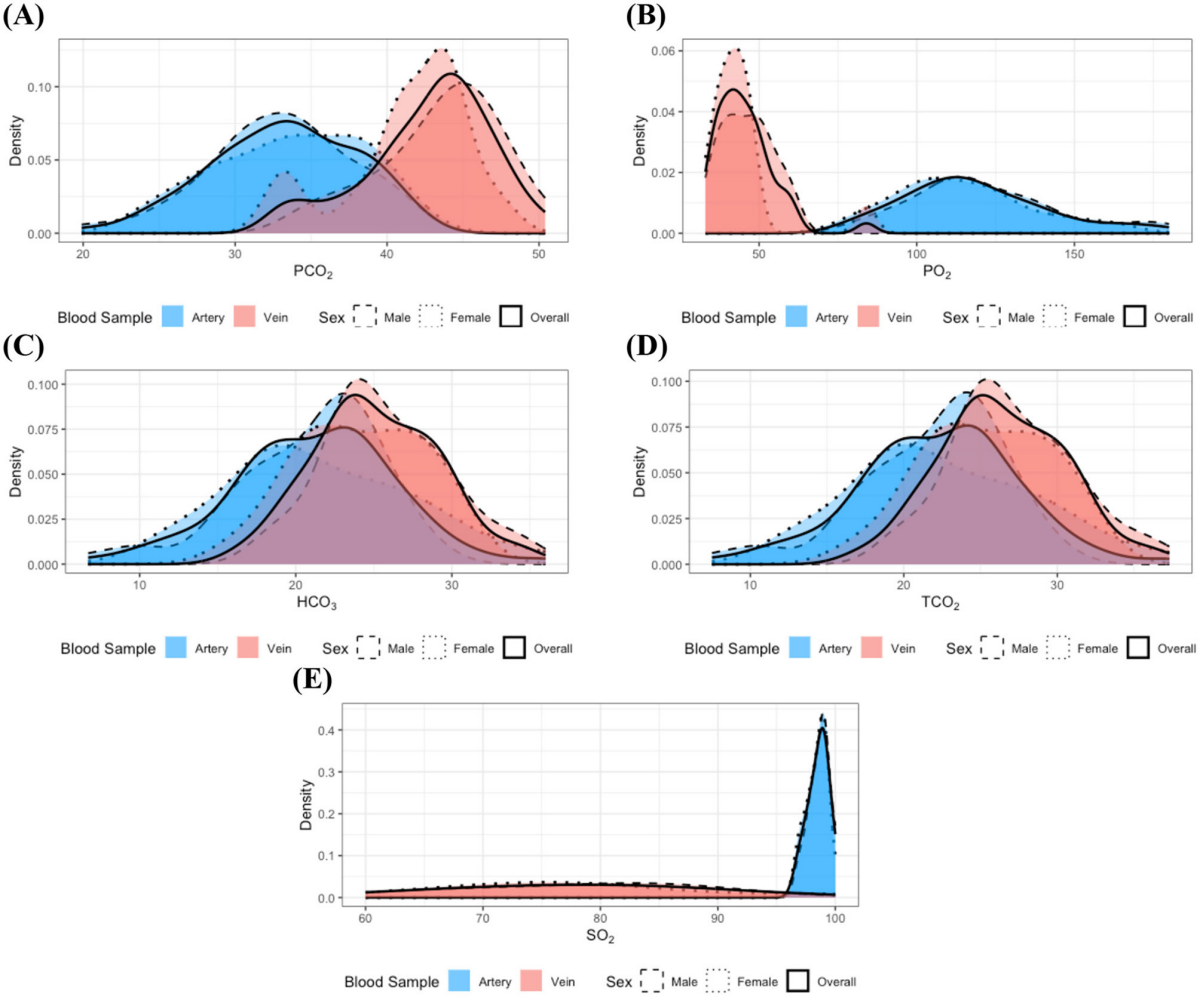
Distributions of the measurements with identified statistically significant distributional differences in the clinically healthy group: **(A)** PCO_2_, **(B)** PO_2_, **(C)** HCO_3_, **(D)** TCO_2_, and **(E)** SO_2_. Dashed, dotted, and solid lines correspond to distributions for male, female, and the combined group, respectively. Red and blue colors denote arterial and venous samples. All distributions were estimated using the kernel density estimation method. The overall distributions obtained by pooling data across sexes closely resemble the distributions of each individual sex group, indicating no significant distributional differences according to sex. In contrast, notable differences in the distributions of measurement are evident between arterial and venous samples.

Figures 1A–E illustrate the distributions of measurements for which statistically significant distributional differences were identified by blood sample type and sex within clinically healthy individuals. The figure distinctly shows varying distribution patterns according to blood sample type. In particular, as shown in Figures 1B, E, PO_2_ and SO_2_ measurements were predominantly higher in arterial samples compared to venous samples, whereas PCO_2_, shown in Figure 1A, was generally elevated in venous samples relative to arterial samples. Furthermore, the variability of these measurements differed between arterial and venous samples; for example, PO_2_ values in venous samples were concentrated within a relatively narrow range compared to those in arterial samples, while SO_2_ values in arterial samples clustered within the 95%−100% but spanned a broader range of 60%−98% in venous samples. These observed distributional differences highlight the necessity for separate clinical interpretations of arterial and venous blood gas parameters, as failing to distinguish between these sample types may introduce bias and lead to artificially widened reference interval estimates.

Although HCO_3_ and TCO_2_ did not achieve statistical significance, their *p*-values closely approached the Bonferroni-adjusted significance level. Moreover, elevated values of HCO_3_ and TCO_2_ (Figures 1C, D) were more frequently observed in venous samples compared to arterial samples. These findings suggest that the distributions of HCO_3_ and TCO_2_ may also differ between blood sample types. Furthermore, examination of Figure 1 indicates that most of the measurements approximate a normal distribution, and the observed similarity in distributions between sexes supports the findings from previous analyses.

**Figure 2:**
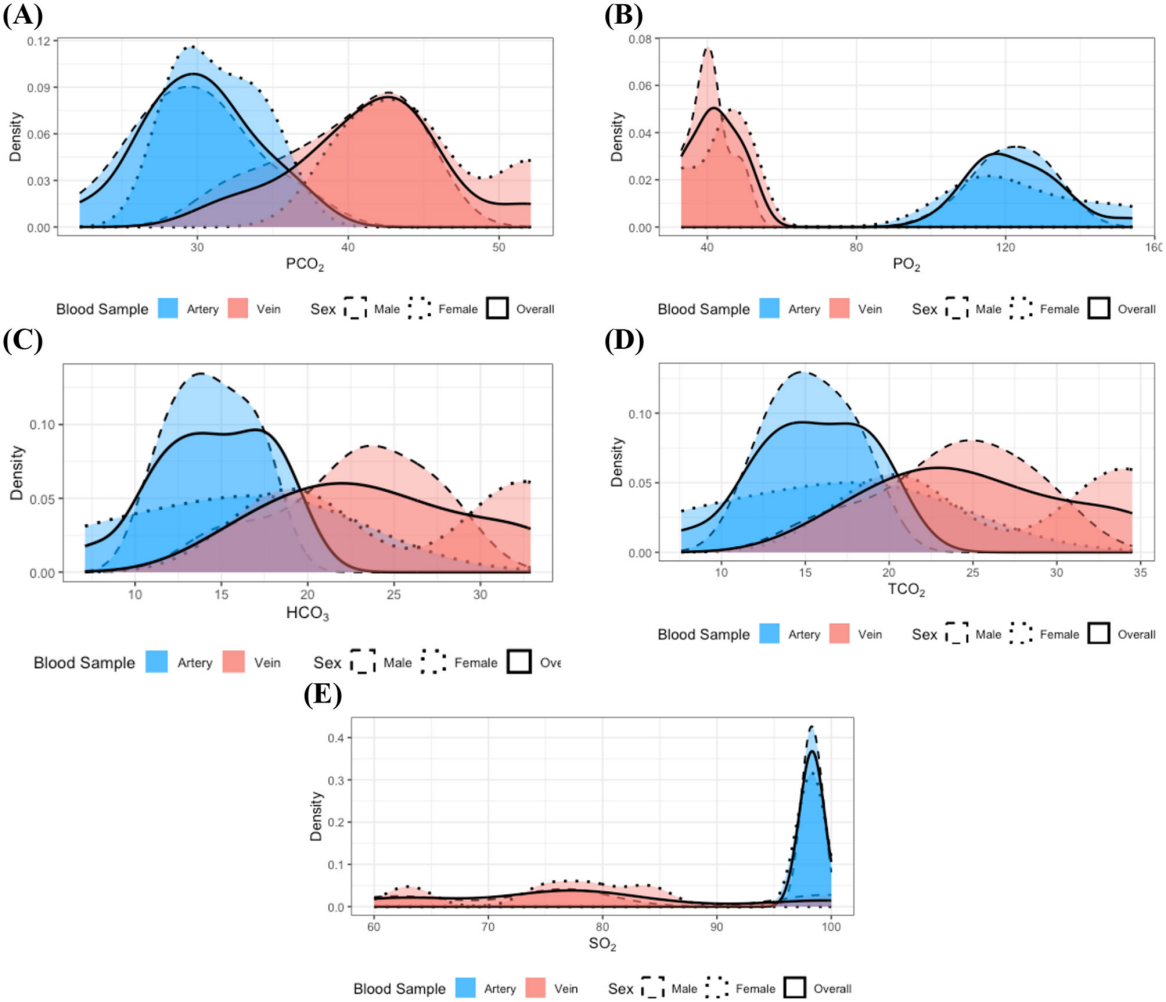
Distributions of the measurements with identified statistically significant distributional differences in the dental disease group: **(A)** PCO_2_, **(B)** PO_2_, **(C)** HCO_3_, **(D)** TCO_2_, and **(E)** SO_2_. Dashed, dotted, and solid lines correspond to distributions for male, female, and the combined group, respectively. Red and blue colors denote arterial and venous samples. All distributions were estimated using the kernel density estimation method. Although clear sex-specific distributional differences are not readily apparent in these measurements, pronounced differences between arterial and venous sample types are evident.

In the study of individuals with dental disease, stratified analyses of measurements by blood sample type and sex did not reveal any statistically significant differences in distribution. This absence of significant findings could be attributed to the small sample size of the dental disease group, which may result in a lack of statistical power to detect true differences. To further investigate this, subsequent integration of data by sex, informed by prior analytical results, led to the identification of statistically significant differences in the distributions of the same measurements as analyzed in the healthy group, depending on blood sample type. Moreover, the results depicted in Figure 2 further corroborate these findings. The distributions of PO_2_ and SO_2_ (Figures 2B, E) indicate that the majority of values observed in venous samples did not overlap with those found in arterial samples. Additionally, PO_2_ measurements in arterial samples exhibited greater variability compared to venous samples, whereas SO_2_ values in venous samples had a larger variance than those in arterial samples. On the other hand, for PCO_2_ (Figure 2A), lower measurement values were more frequently observed in arterial samples compared to venous samples. Furthermore, high measurement values of HCO_3_ and TCO_2_ constituted a larger proportion of observations in venous samples than in arterial samples (see Figures 2C, D).

**Figure 3:**
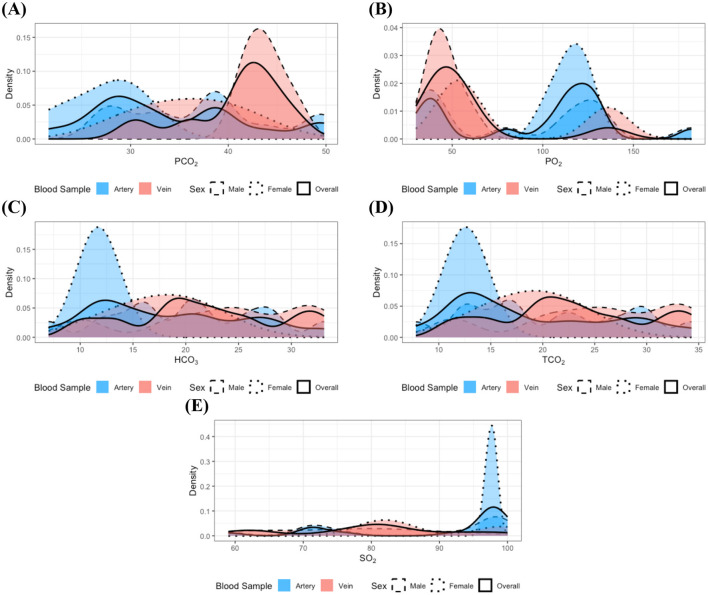
Distributions of the measurements with identified statistically significant distributional differences in the gastrointestinal disease group: **(A)** PCO_2_, **(B)** PO_2_, **(C)** HCO_3_, **(D)** TCO_2_, and **(E)** SO_2_. Dashed, dotted, and solid lines correspond to distributions for male, female, and the combined group, respectively. Red and blue colors denote arterial and venous samples. All distributions were estimated using the kernel density estimation method. Although the limited sample size in the gastrointestinal disease group makes it challenging to clearly delineate the distributional patterns, potential differences according to blood sample type can still be inferred.

In the analysis of individuals classified within the gastrointestinal disease group, no statistically significant differences were observed in the distributions of all measurements, even following the integration of samples by sex. Nevertheless, it is important to remain cautious regarding measurements previously identified as exhibiting distributional differences because of the relatively small sample size within this group. Consistent with the findings in the clinically healthy and dental disease groups, the distributions of PO_2_ and SO_2_ (Figures 3B, E) appeared bimodal, with each mode corresponding to a distinct blood sample type; a similar bimodal distribution was also observed for PCO_2_ (Figure 3A). In contrast, the distributions of HCO_3_ and TCO_2_ (Figures 3C, D) did not exhibit clear bimodality but were characterized by a greater concentration of lower values in arterial samples. These observations suggest that distributional differences in these measurements may still exist in the gastrointestinal disease group.

The sample sizes in the musculoskeletal and urologic disease groups were insufficient to achieve the statistical power necessary for the Kolmogorov-Smirnov test to detect significant distributional differences. Despite this limitation, the previous findings suggest a hypothesis that the distributions of several measurements—specifically PCO_2_, PO_2_, HCO_3_, TCO_2_, and SO_2_–may vary based on blood sample types in these disease groups. Accordingly, subsequent analyses were conducted with data stratified by blood sample type for these specific measurements in the musculoskeletal and urologic disease groups.

### 3.2 Analysis of reference interval

The 95% reference intervals for blood gas parameters, along with summarized descriptive statistics, are presented in Table 3. The reference intervals for arterial samples established in the current study closely correspond to those previously reported by (10). Minor observed discrepancies may be attributed to variations in samples and differences in the statistical methods employed to estimate the reference intervals. For example, in this study, the reference interval for arterial PCO_2_ was estimated using the parametric approach based on observation suggesting that PCO_2_ followed a normal distribution and the sample size was <120. In contrast, the non-parametric approach was utilized in (10). A notable advancement of the present study compared to previous investigations is the establishment of reference intervals for both arterial and venous blood samples. Providing these intervals confers practical advantages by diminishing the requirement for selectively targeting specific blood vessels during sampling, thus simplifying clinical procedures.

**Table 3:** 95% reference intervals and summary statistics for blood gas parameters in the clinically healthy group.

**Parameter**	**Blood vessel**	**Mean**	**Standard deviation**	**Reference interval**
pH	Artery/Vein	7.40	0.08	7.22–7.55
PCO_2_ (mmHg)	Artery	33.23	4.79	23.85–42.62
	Vein	42.72	4.13	34.61–50.82
PO_2_ (mmHg)	Artery	118.72	23.28	79.47–169.12
	Vein	45.65	9.56	33.29–70.00
Na (mmol/L)	Artery/Vein	142.56	2.99	136.20–147.80
K (mmol/L)	Artery/Vein	4.40	0.53	3.50–5.60
Ca (mg/dL)	Artery/Vein	1.69	0.11	1.46–1.89
Cl (mmol/dL)	Artery/Vein	103.46	5.58	92.25–115.75
Hct (%)	Artery/Vein	40.51	4.22	31.52–49.80
HCO_3_ (mmol/L)	Artery	21.31	4.85	11.81–30.81
	Vein	24.86	4.22	16.60–33.13
	Artery/Vein	23.19	4.34	13.34–31.20
BB (mmol/L)	Artery/Vein	−2.21	5.84	−13.96–8.52
BE (mmol/L)	Artery/Vein	−2.45	6.58	−15.18–9.66
TCO_2_ (mmol/L)	Artery	22.33	4.93	12.67–31.98
	Vein	26.17	4.30	17.75–34.60
A-a gradient (mmHg)	Artery	−8.79	22.22	−47.45–29.88
HGB (g/dL)	Artery/Vein	12.60	1.26	10.20–15.45
SO_2_ (%)	Artery	98.58	0.87	96.65–100.00
	Vein	77.83	9.58	59.05–96.61
AG (mmol/L)	Artery/Vein	21.18	6.92	6.90–35.55
iCal (mmol/L)	Artery/Vein	1.69	0.10	1.46–1.85

### 3.3 Comparison of reference intervals between healthy and disease groups

**Figure 4:**
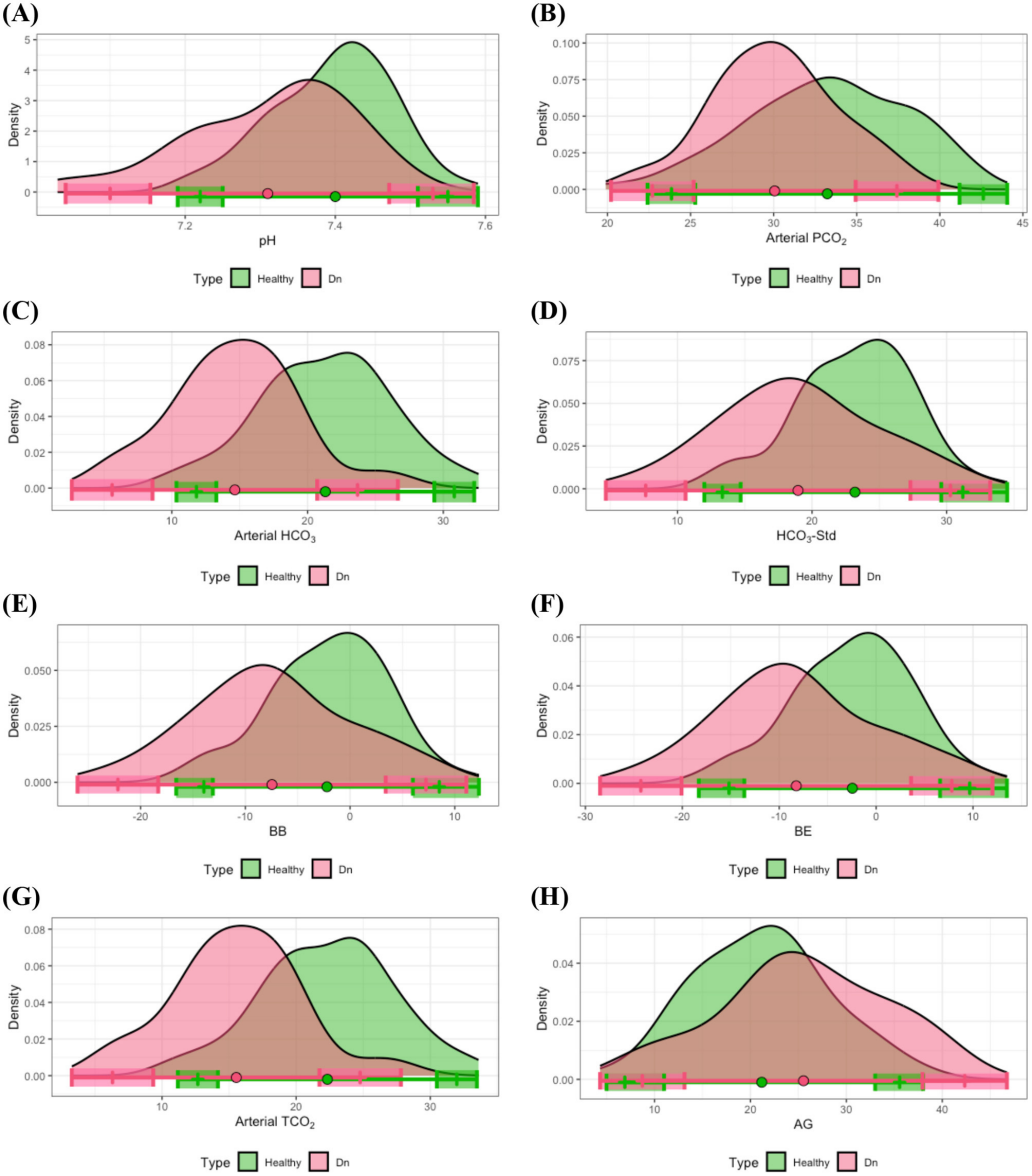
Distributional comparisons and reference intervals for clinically healthy and dental disease groups: **(A)** pH, **(B)** arterial PCO_2_, **(C)** arterial HCO_3_, **(D)** HCO_3_-Std, **(E)** BB, **(F)** BE, **(G)** arterial TCO_2_, and **(H)** AG. Colored dots represent the mean values for each group, while shaded regions denote the 90% CIs of the respective reference limits. Notably, at least one of the 90% CIs for either the lower or upper reference interval limits is non-overlapping between the healthy and dental disease groups, signifying significant differences in reference intervals between these groups.

The estimated disease-associated reference intervals are detailed in Table 4. Analysis demonstrated that although some overlap existed between the heathy-associated and disease-associated reference intervals, several notable differences were identified. Specifically, both the lower and upper limits of the reference interval for pH in dental disease group were lower compared to those derived from the healthy group. Statistical analysis using a *t*-test along with Levene's test confirmed significant differences between the means of pH for the healthy and dental groups (*p*-value < 0.001). Moreover, the lower limit of the disease-associated reference interval was significantly lower compared to that of the healthy reference interval in terms of the 90% CIs. In addition, subjects within the dental disease group more frequently exhibited lower pH values relative to their healthy counterparts; specifically, ~23% of individuals exhibited pH measurements falling below the established lower reference limit for the clinically healthy group (see Figure 4A).

**Table 4:** 95% reference intervals for blood gas parameters according to disease groups.

**Parameter**	**Blood vessel**	**Disease-associated reference interval**			
		**Dn**	**GI**	**Mus**	**Uro**
pH	Artery/Vein	7.10–7.53	7.08–7.55	7.35–7.55	7.28–7.40
PCO_2_ (mmHg)	Artery	22.70–37.42	18.31–50.33	22.69–38.06	16.89–58.23
	Vein	35.56–47.47	29.52–50.55	29.77–53.15	-
PO_2_ (mmHg)	Artery	97.37–152.30	13.39–219.49	73.04–142.46	28.50–156.30
	Vein	30.53–115.65	34.51–203.69	15.28–62.72	-
Na (mmol/L)	Artery/Vein	135.01–149.65	133.10–148.38	139.48–146.02	140.53–146.47
K (mmol/L)	Artery/Vein	3.49–5.23	3.09–5.38	3.03–5.66	2.96–5.90
Ca (mg/dL)	Artery/Vein	1.50–1.92	1.38–1.85	1.53–1.82	1.46–1.85
Cl (mmol/dL)	Artery/Vein	94.33–111.87	84.32–111.92	86.33–119.01	96.48–117.52
Hct (%)	Artery/Vein	28.74–50.59	24.20–54.92	32.53–38.47	34.19–48.81
HCO_3_ (mmol/L)	Artery	5.60–23.68	3.02–32.48	1.79–39.51	9.53–31.83
	Vein	12.36–35.14	7.00–36.25	25.95–27.65	-
	Artery/Vein	7.64–30.28	6.23–32.57	20.56–28.94	14.11–27.45
BB (mmol/L)	Artery/Vein	−22.17–7.25	−23.84–10.35	−7.93–11.24	−14.17–3.90
BE (mmol/L)	Artery/Vein	−24.28–7.81	−25.88–11.36	−9.07–12.18	−16.36–5.06
TCO_2_ (mmol/L)	Artery	6.31–24.77	2.86–33.75	2.47–40.68	10.11–33.57
	Vein	13.46–36.58	8.13–37.55	26.99–29.15	-
A-a gradient (mmHg)	Artery	−22.82–9.05	−61.18–81.41	−25.77–33.77	−28.72–49.52
HGB (g/dl)	Artery/Vein	8.91–15.71	5.94–15.89	10.08–11.99	10.61–15.16
SO_2_ (%)	Artery	96.73–99.88	64.98–100.00	96.40–99.60	95.28–100.00
	Vein	51.93–100.00	56.56–97.69	41.39–100.00	-
AG (mmol/L)	Artery/Vein	8.73–42.34	7.86–45.07	5.97–52.62	18.11–24.89
iCal (mmol/L)	Artery/Vein	1.50–1.87	1.29–1.86	1.61–1.82	1.42–1.78

A dash (“–”) indicates that a reference interval could not be established due to insufficient data.

Further differences in reference intervals for the dental disease group were observed in arterial PCO_2_, arterial HCO_3_, HCO_3_-Std, BB, BE, arterial TCO_2_, and AG. *T*-tests substantiated that the mean values of these parameters significantly differed from those in the healthy group, with all corresponding *p*-values < 0.01. Additionally, significant differences were noted in at least one of the lower or upper limits of the reference intervals between healthy and dental groups. As illustrated in Figures 4B–H, individuals within the dental disease group commonly exhibited lower values for arterial PCO_2_, arterial HCO_3_, HCO_3_-Std, BB, BE, and arterial TCO_2_ compared to healthy subjects, whereas AG levels were frequently higher in dental disease subjects than in their healthy counterparts.

**Figure 5:**
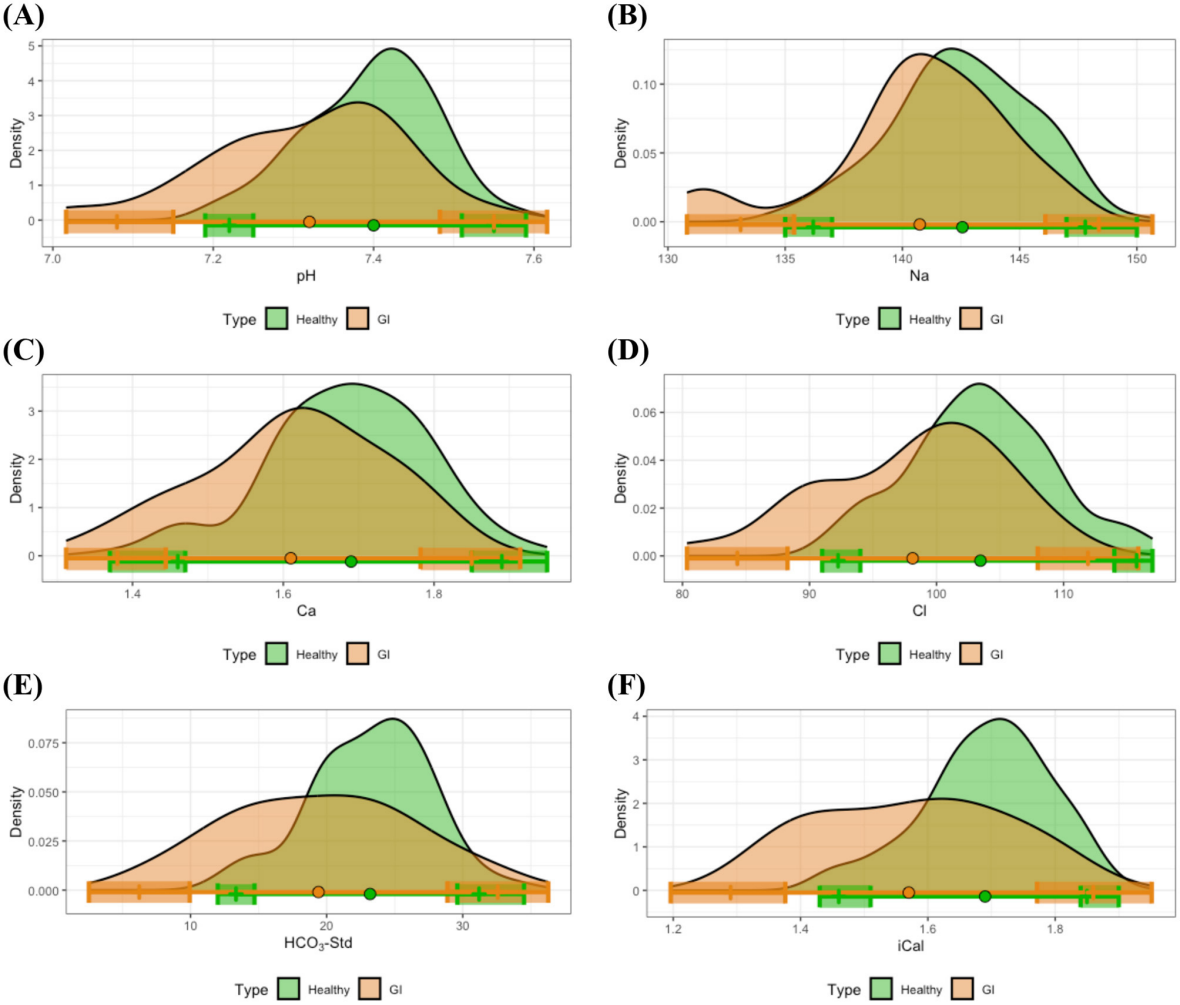
Distributional comparisons and reference intervals for clinically healthy and gastrointestinal disease groups: **(A)** pH, **(B)** Na, **(C)** Ca, **(D)** Cl, **(E)** HCO_3_-Std, and **(F)** iCal. Colored dots represent the mean values for each group, while shaded regions denote the 90% CIs of the respective reference limits. For the majority of measurements, at least one of the 90% CIs for either the lower or upper reference interval limits does not overlap between the healthy and gastrointestinal disease groups, indicating statistically significant differences in reference intervals between these groups.

Another notable observation concerned sodium levels. Specifically, in the gastrointestinal disease group, the lower limit of the reference interval for Na was substantially lower compared to the healthy group (Figure 5B). A *t*-test indicated a statistically significant difference between the means of these groups, and the distribution of Na in the gastrointestinal disease group demonstrated a heavy left tail, suggesting that gastrointestinal disease subjects frequently presented with lower Na concentrations. Similarly, the lower limits of the reference intervals for pH, Ca, Cl, HCO_3_-Std, and iCal were also reduced in the gastrointestinal disease group compared to healthy controls. The result from the *t*-tests, combined with the comparison of 90% CI of the reference limits and the distribution patterns shown in Figures 5A, C–F, indicated that gastrointestinal disease patients commonly presented with reduced levels of pH, Ca, Cl, HCO_3_-Std, and iCal levels relative to healthy individuals.

**Figure 6:**
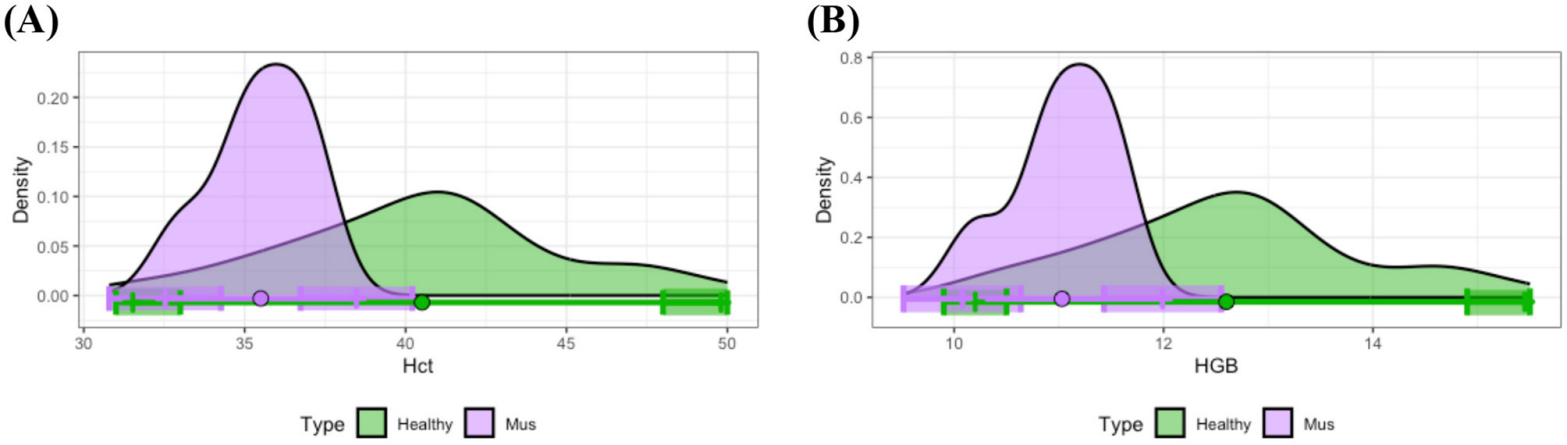
Distributional comparisons and reference intervals for clinically healthy and musculoskeletal disease groups: **(A)** Hct and **(B)** HGB. Colored dots represent the mean values for each group, while shaded regions denote the 90% CIs of the respective reference limits. For each parameter, at least one of the 90% CIs for either the lower or upper reference interval limits does not overlap between the healthy and musculoskeletal disease groups, thereby demonstrating statistically significant differences in reference intervals between these groups.

In contrast to other disease groups, the musculoskeletal disease group exhibited distinct differences from the healthy group regarding Hct and HGB. Specifically, as depicted in Figures 6A, B, most individuals in the musculoskeletal disease group presented with lower levels of Hct and HGB compared to the means observed in healthy individuals. Examination of the 90% CIs for reference limits demonstrated significantly reduced upper limits for Hct and HGB within the musculoskeletal disease group relative to healthy controls. Statistical analysis using *t*-tests further confirmed significant differences between the means of these measurements for the musculoskeletal and healthy groups.

In the urological disease group, no statistically significant differences were identified in measured parameters compared to the healthy group. This is likely attributed to the small sample size of rabbits with urological disease, resulting in reduced statistical power for detecting group differences.

## 4 Discussion

This study established reference intervals for venous and arterial blood gas analysis parameters in both healthy and diseased rabbits. Rabbits with dental, gastrointestinal, musculoskeletal, and urological disease models were evaluated and compared to the healthy control group. Distributional analysis according to the blood sample revealed higher PO_2_ and lower PCO_2_ in the arterial blood. These findings align with previous veterinary reports involving dogs, cats, and rabbits (3, 4). The observed differences are attributed to gas exchange processes occurring in the pulmonary circulation, leading to higher PO_2_ and lower PCO_2_ values in arterial blood (3). Specifically, the established reference intervals of PCO_2_ for healthy rabbits in this study, with the mean values of 33.23 mmHg in arterial blood and 42.72 mmHg in venous blood, closely matched previously reported values (4, 10).

Rabbits diagnosed with dental disease exhibited lower levels of pH, PCO_2_, HCO_3_, HCO_3_-Std, BB, BE, and TCO_2_ compared to clinically healthy rabbits, indicative of metabolic acidosis. Frequently, values below the established normal ranges were observed within the dental disease group. Dental disease is prevalent in rabbits and commonly results in clinical signs such as anorexia, hypersalivation, gastrointestinal stasis, and the potential spread of periodontal infection to adjacent anatomical areas (22, 23). Bicarbonate loss following hypersalivation in dental disease could explain the results in this study, as metabolic acidosis following hypersalivation has been demonstrated in equine and bovine studies (24). Additionally, anorexia induces metabolic acidosis in rabbits (9). Anorexia causes hypoglycemia, which results in lipolysis through beta-oxidation, producing ketone bodies, which cause ketoacidosis. Also, lipid deposits in the liver develop hepatic lipidosis (23, 25).

Oral flora is maintained by acid-base adaptation, and dental diseases are associated with disturbances in oral pH (26). However, whether acid-base disturbances in oral flora caused by dental disease can precipitate systemic disturbances in the bloodstream remains unclear. In studies involving humans and dogs, the association between dental disease and systemic inflammatory response syndrome (SIRS) has been speculated, wherein the translocation of periodontal bacteria into the bloodstream induces systemic inflammation (27). SIRS is frequently accompanied by severe metabolic acidosis (27). However, the extent of the reduction of pH and bicarbonate in this study was not as extreme as that of SIRS.

Previous research has demonstrated a plausible connection between periodontal disease and renal pathology. In canine studies, dental disease has been identified as a possible risk factor for the development of chronic kidney disease (28). Furthermore, clinical reports in human medicine have indicated a potential link between dental disease and renal tubular acidosis (29). Renal tubular function plays a pivotal role in maintaining electrolyte and acid-base homeostasis through the regulated reabsorption and excretion of hydrogen ions and bicarbonate. Consequently, dental diseases that impact renal function can hypothetically result in alterations in blood gas parameters due to impaired acid-base and electrolyte regulation. However, evidence supporting a relationship between dental disease and renal dysfunction in rabbits remains lacking.

The findings of the present study contrast with those reported in previous literature. Earlier research demonstrated that rabbits with advanced dental disease exhibited reduced serum calcium concentrations (22). In addition, rabbits diagnosed with acquired dental disease showed evidence of hypocalcemia accompanied by elevated serum parathyroid hormone levels (22). These alterations have been attributed to osteodystrophy and impaired calcification processes affecting both bone and dental tissues, which are implicated in the pathogenesis of acquired dental disease in rabbits (22). Consistently, studies conducted in humans and rats have also reported associations between dietary calcium intake and dental health, wherein inadequate calcium intake was linked to an increased incidence of periodontal disease (30).

Several studies have investigated the sequelae of gastrointestinal disease in rabbits. Diarrhea in rabbits is commonly associated with dehydration and hypokalemia due to gastrointestinal fluid losses (23). Additionally, appendicitis in rabbits has been reported to cause hypocalcemia and hypoglycemia (31). Gastrointestinal stasis—also referred to as gastrointestinal syndrome—has been linked to metabolic acidosis (9). Hyponatremia has been identified as a negative prognostic indicator in gastrointestinal syndrome, and both electrolyte and acid-base imbalances have been shown to progressively worsen in the absence of timely medical intervention (9). In this study, acid-base imbalance within the gastrointestinal disease group was evidenced by decreased values of both pH and standard bicarbonate. In addition, rabbits in the gastrointestinal disease group demonstrated significantly lower serum concentrations of sodium, chloride, and calcium when compared to the clinically healthy control group. These findings regarding electrolyte derangements are consistent with those reported in previous studies (9, 31).

One of the potential mechanisms contributing to the development of metabolic acidosis in rabbits with gastrointestinal disease is hepatic lipidosis. Hepatic lipidosis is a common complication of gastrointestinal stasis (25). Also, any gastrointestinal tract disease causing anorexia develops hepatic lipidosis, especially for obese rabbits or rabbits having high-fat diets. In the absence of adequate nutritional intake, free fatty acids are mobilized and metabolized in the liver as alternative energy substrates to glucose and volatile fatty acids. This metabolic shift leads to the production of ketone bodies, which, when elevated, result in ketoacidosis (23).

However, small animals, including dogs and cats, have several different blood gas values that result from gastrointestinal disease. Acid-base disturbance related to the gastrointestinal system depends on the primary manifestation of the disease. Diarrhea results in metabolic acidosis by bicarbonate loss, whereas vomiting results in metabolic alkalosis due to the loss of hydrogen ions (32, 33). The gastrointestinal tract, especially the jejunum, is highly permeable to absorb sodium, potassium, and chloride (32). Therefore, hyponatremia, hypokalemia, and hypochloremia may frequently occur as a consequence of loss throughout the gastrointestinal tract (2). However, gastrointestinal disease rarely alters calcium, which is mainly associated with renal, endocrine disease, or malignancy (34).

The pathophysiological mechanisms linking musculoskeletal disease to acid-base or electrolyte imbalances remain poorly understood. Diagnostic evaluation of musculoskeletal disorders generally relies on specific biomarkers rather than blood gas parameters, as the musculoskeletal system is not primarily involved in the regulation of acid-base balance and electrolyte homeostasis, unlike the gastrointestinal, urologic, and respiratory systems. In equine medicine, a field in which musculoskeletal disorders are particularly prevalent, commonly utilized biomarkers include creatinine kinase and serum aspartate aminotransferase (35). Studies with musculoskeletal disease models in rabbits revealed several pathognomonic serum biomarkers, such as C-telopeptide of type II collagen, bone morphogenic protein 2, and cartilage oligomeric matrix protein (36).

Therefore, it is logical to conclude that the results of the musculoskeletal disease model in this study are clinically insignificant. The musculoskeletal disease model in this study showed lower levels of Hct and HGB compared to healthy rabbits. The observed decrease in Hct may indicate anemia or fluid shifts; however, its specific clinical implications in musculoskeletal disorders remain unclear and require further investigation. The complete blood count analyzer remains the golden standard for hemoglobin measurement, as previous studies have indicated that point-of-care blood gas analyzer tends to underestimate hemoglobin levels compared to standard measurements (37). Thus, reliance solely on blood gas values is not recommended for decisions regarding transfusion or estimating blood loss (37). Given these findings, further research is necessary to determine if these hematologic changes are directly related or clinically meaningful in musculoskeletal disorders.

Research on acid-base disturbances or electrolyte imbalances in musculoskeletal disease has indicated that blood gas derangements are usually risk factors or causes rather than consequences. Studies in rabbits report higher potassium concentrations in venous compared to arterial blood (23). Metabolic causes of muscle weakness in rabbits include hypocalcemia and hypokalemia (23, 25). Rabbits on diets with unbalanced calcium-phosphorus ratios may develop nutritional osteodystrophy, driven by hypocalcemia and subsequent secondary hyperparathyroidism, leading to increased bone resorption, a known mechanism in metabolic bone disease pathogenesis (22, 23, 38). Certain skeletal disorders, such as osteolytic bone tumors, can induce electrolyte imbalance and hypercalcemia (38).

In human medicine, systemic acidosis adversely impacts bone and skeletal muscle, activating osteoclasts to increase bone resorption and inhibiting osteoblasts, reducing mineral deposition (39). Additionally, metabolic acidosis impairs skeletal muscle function and protein turnover, contributing to muscle wasting (40). Equine studies also document acid-base and electrolyte disturbances associated with muscular diseases, such as atypical myopathy, characterized by lactic acidosis, hyponatremia, hypochloremia, hyperkalemia, hyperphosphatemia, and hypocalcemia due to rhabdomyolysis (41). Exercise-induced electrolyte disturbances in equine species, such as hypokalemia from sweating and hyperkalemia from muscular potassium release, are also well-documented (23). Nevertheless, evidence regarding similar blood gas disturbances in musculoskeletal diseases, specifically in rabbits, remains limited.

Rabbits have distinctive urinary tract characteristics compared to other species. Calcium homeostasis in rabbits highly depends on urinary excretion, while dietary calcium absorption occurs primarily through the gastrointestinal tract. Rabbits exhibit higher and broader blood calcium ranges due to dietary calcium being absorbed passively, independent of Vitamin D3-mediated active transport typical in other mammals (38). Calcium excretion in rabbits predominantly occurs as calcium carbonate precipitate in urine, proportional to dietary intake. This renal excretion process is regulated by parathyroid hormone and 1,25-dihydroxyvitamin D (23, 38). However, rabbits exhibit lower renal tubular calcium reabsorption rates than dogs and humans (38).

Furthermore, rabbits possess limited renal capabilities in electrolyte and acid-base regulation. Their capacity to excrete hydrogen ions via urine is restricted due to two factors: rabbit renal tubular epithelium lacks carbonic anhydrase, which is essential for converting carbon dioxide to bicarbonate and subsequently excreting hydrogen ions; additionally, rabbits exhibit limited glutamine deamination, which is crucial for ammonium ion-mediated hydrogen ion excretion. Consequently, rabbits are highly vulnerable to metabolic acidosis (23).

In this study, rabbits with urological disease did not exhibit statistically significant differences compared to healthy controls, which is inconsistent with previous research (23, 25). Rabbit with acute kidney injury and chronic kidney disease results in hypercalcemia (25). Typically, chronic renal failure in rabbits reduces calcium excretion without impacting calcium absorption, thus causing hypercalcemia; however, this phenomenon was not observed in the current study. Chronic renal failure in rabbits may also lead to hyponatremia and hyperkalemia due to impaired renal concentrating ability, wherein rapid urine flow through renal tubules hinders effective sodium-potassium exchange, reducing potassium excretion (23).

Comparable electrolyte and acid-base disturbances occur in other species, such as dogs and cats, with urinary tract disorders. Typically, renal diseases induce metabolic acidosis through impaired bicarbonate reabsorption and elevated chloride levels, along with potential disturbances in sodium, potassium, and calcium concentrations (32).

This study is novel to analyze arterial and venous samples for blood gas analysis in the context of various breeds and disease models. Reference intervals were established for blood gas and electrolyte parameters in rabbits diagnosed with dental, gastrointestinal, urological, and musculoskeletal diseases, and these were systematically compared with values from a clinically healthy control group. This comparative approach enables the evaluation of disease-specific trends in acid-base and electrolyte disturbances. Current literature provides limited evidence regarding blood gas abnormalities associated with specific disease states in rabbits (9, 42). The clinical significance of the present study lies in its contribution to understanding the impact of various pathological conditions on the blood gas values of rabbits. This study will benefit in providing diagnostic clues and possible prognostic indicators for various rabbit diseases.

This study also provides validation for the use of a point-of-care blood gas analyzer in rabbits. Blood gas analysis serves as an essential diagnostic tool for assessing critically ill patients, as severe acid-base and electrolyte imbalances can have life-threatening consequences. Additionally, deviations in blood gas parameters may offer valuable insights into underlying pathological conditions. The utility of a point-of-care device lies in its capacity to deliver rapid, clinically actionable results. Previous studies have demonstrated that this point-of-care analyzer is validated for canine, feline, and bovine patients (13). However, data supporting its application in rabbits has been limited. Prior research in rabbits reported poor agreement between the point-of-care device and in-house reference analyzer, except for pH measurements, which remained consistent between both platforms (42). In the present study, blood gas values obtained from healthy rabbits using the i-Smart 300 VET Blood Gas Analyzer were consistent with previously reported reference data from an in-house analyzer. These findings suggest that the point-of-care device is reliable for the rabbit species.

There are several limitations in this study. First, the evaluation of disease groups had limitations. No specific diseases were stated for each group. Even diseases in the same body system can result in different blood gas values, and this study did not reveal those detailed variations within the same disease model. The clinical outcome of cases in the diseased group was also not stated. Critical disease states like sepsis, which causes significant changes in blood gas values, cannot be determined only with a single blood test method. Furthermore, this study compared disease and healthy groups regarding reference range, which does not examine individual values. While reference intervals provide a framework for evaluating general trends and parameter distributions, they do not allow for detailed analysis of individual variability or the detection of specific increases or decreases in individual values.

Second, the composition of the study sample presents certain limitations. The sample size and sex distribution were not uniform across the different disease groups, which may have introduced statistical bias. In particular, certain disease types, such as urologic disease, were represented by very small sample sizes. This limitation may have substantially reduced the statistical power to detect significant differences or distinct distributional patterns within their subgroups, as the sensitivity of statistical methods to discern such differences is largely dependent on the adequacy of sample size. Additionally, although rabbits from multiple breeds were included in the study, their representation was uneven across disease groups, and the analysis did not account for potential breed-related variation in blood gas parameters. Given that breed-specific physiological differences may exist, the uneven distribution of breeds could have introduced bias in the estimated reference intervals.

Finally, the selection of disease models in this study was limited. Arterial blood gas analysis is routinely used for monitoring anesthetic and ventilatory status, as well as for assessing respiratory function (10, 11). However, this study did not include a respiratory disease model. Blood samples were collected under light anesthesia, but this study did not consider the procedural effect of anesthesia or sedation on the blood gas value. Isoflurane, an inhalant anesthetic used in this study, is well known for inducing cardiovascular and respiratory depression in veterinary species, including rabbits (43). Due to isoflurane-induced hypoventilation, related blood gas values are likely to vary, potentially misleading the comparison between disease and healthy groups. Therefore, further research with more cases, equal distribution of cases within each group, other disease models, and anesthesia/sedation models known to affect blood gas is recommended.

## Data Availability

The raw data supporting the conclusions of this article will be made available by the authors, without undue reservation.
